# Structural and Thermal Stability of Polycarbonate Decorated Fumed Silica Nanocomposite via Thermomechanical Analysis and *In-situ* Temperature Assisted SAXS

**DOI:** 10.1038/s41598-017-08122-7

**Published:** 2017-08-09

**Authors:** Ramdayal Yadav, Minoo Naebe, Xungai Wang, Balasubramanian Kandasubramanian

**Affiliations:** 1 Deakin University, Institute for Frontier Materials (IFM), Geelong, Australia; 2 Department of Materials Engineering, Defence Institute of Advanced Technology, Ministry of Defence, Girinagar, Pune 411025 India

## Abstract

The inorganic and organic nanocomposites have enticed wide interest in the field of polymer-based composite systems to augment their physiochemical properties like mechanical strength and electrical conductivity. Achieving interfacial interaction between inorganic filler and polymer matrix is a recurring challenge, which has significant implications for mechanical properties of nanocomposites. In this context, the effect of “interfacial zone” on structural and thermal attributes of the melt blended pristine polycarbonate and polycarbonate (PC) decorated fumed silica nanocomposite have been examined from ambient temperature to the glass transition temperature. Thermomechanical characterization and *in-situ* temperature assisted small angle X-ray scattering technique (SAXS) were used for contemplating quantitative and qualitative molecular dynamics of developed nanocomposites. Though, the FT-IR spectra have demonstrated some extent of interaction between inorganic and organic groups of composite, the reduced glass transition temperature and storage modulus was ascertained in DMA as well as in DSC, which has been further confirmed by *in-situ* temperature assisted SAXS. It is envisioned that the utilization of *in-situ* SAXS in addition to the thermomechanical analysis will render the qualitative and quantitative details about the interfacial zone and its effect on thermal and mechanical properties of nanocomposite at varying temperature conditions.

## Introduction

High-performance polymer nanocomposites have numerous application of fundamental to applied research in conventional engineering. These nanocomposites are reinforced with variable size fillers including carbon-based materials (Nanotube, Nanofibers), layered silicates (e.g. montmorillonite, saponite), nanoparticles of metals (e.g. Au, Ag), metal Oxides (e.g. TiO_2_, ZnO)^1^. It is generally accepted that the interface zone, formed between polymer and nanoparticles plays decisive role in the performance of nanocomposite because it impels physical and some cases chemical properties of composite systems^2^. In this context, SiO_2_ nanoparticles has been recognized as a prominent nanofiller for substantial enhancement in thermal and mechanical properties of polymer nanocomposite by the virtue of its intriguing aspect of interface zone modification by substituting its surface silanol group by multifunctional and compatible active agents^3–5^. Additionally, fumed silica has also evinced its ability to chemically connect with polymer precursor and its functional group for further utilizing in various processes such as sol-gel process, *in-situ* surface-initiated polymerization, photo-polymerization and surface initiated polymerization^6^. In this context, group of Bikiaris *et al*. have extensively exploited the fumed silica based polymer nanocomposites by utilizing range of polymer including poly(vinyl pyrrolidone), chitosan, poly (vinyl alcohol), poly (ethylene succinate), poly (butylene succinate) etc to evaluate the effect of silica filler on thermal and dynamic mechanical behaviour of the polymers^7–10^. They have demonstrated that the extent of the property enhancement largely depends on the interaction of silanol group of fumed silica and the functional group of polymer chain and type of interaction (hydrogen bonding, covalent bond or branching or crosslinking) confide in processing method i.e. solvent evaporation, *in-situ* polymerization, condensation polymerization. The group has concluded that the interaction of fumed silica with polymer chain play a prominent role in exemplifying the thermal mechanical and rheological properties of any polymeric systems.

Polycarbonate (PC) has been broadly investigated as an imperative polymer for applied and fundamental research due to its numerous appreciable properties such as high strength, ductility with high glass transition temperature and optical transparency^11^. Consequently, PC has been exhaustively exploited in application like electronic appliances, automobile, architecture and aerospace. Pryde *et al*. have demonstrated that polycarbonate under the condition of humidity and temperature is conducive to the rapid hydrolysis if it do not contain hydrolytic stabilizers^12, 13^. In another study Devis *et al*. manifested that polycarbonate possesses a considerable degree of thermal stability, but in the evacuated system, it undergoes little decomposition below^14^. Thermal and mechanical attributes of polycarbonate have been investigated in conjunction with various nano additives like polyhedral oligomeric silsesquioxane (POSS)^15^, Graphene^16^ and montmorillonite^17^ but Feng *et al*. delineated that PC/silica nanocomposite possess ability for potential application in the field of consumer electronics like mobile phone shells, aircraft and automobile sector. They have demonstrated that such nanocomposite exhibits unparalleled thermostability with enhanced immobilization of nanoparticles in polymer chains or matrix^18^. Motaung *et al*. reported analogous results and investigated thermal and mechanical behaviour of polycarbonate with systematic increase (1 to 5 wt%) of silica nanoparticles concentration in polymer matrix and concluded that 2 wt% silica rendered higher thermal stability compared to 5 wt% of silica concentration due to the silica agglomeration at higher loading condition^19^. In contrast to these abstractions, Han *et al*. reported that thermal and mechanical properties of polycarbonate can be augmented by developing long chain branched polycarbonate (LCB-PC) by utilizing linear PC as precursor via gamma radiation technique. They have demonstrated that availability of linear fraction and LCB fraction leads to the bimodal or tri-model distribution of mass which widely affects the thermal, mechanical and rheological attributes of PC at some extent^20^.

In order to characterize, thermal stability of the polycarbonate-based composites, various methodologies like thermogravimetry analysis, differential thermal analysis, dielectric, thermal analysis and dynamic mechanical analysis, etc. have been extensively exploited but the simultaneous evolution of structural and thermal integrity is an intricate phenomenon. Whereas, small angle X-ray technique has been reported as another effective tool to discern the structural variation in polycarbonate based system, including the effect of annealing temperature, craze initiation, effect of solvents, composite and blends, but there are only few literatures available which deal with the temperature assisted *in-situ* SAXS characterization of polymeric artefacts^21–27^. In this contribution, we have demonstrated the utilization of temperature assisted *in-situ* small angle x-ray scattering technique followed by dynamic mechanical analysis (DMA), Differential scanning calorimetry (DSC), thermal gravimetric analysis (TGA), Fourier transform infrared spectroscopy FT-IR, Tensile and Impact Testing to probe repercussion of “interfacial zone” on thermal and structural stability of polycarbonate/Fumed Silica nanocomposite (PC/FS) by qualitatively and quantitatively. We have speculated that bulk PC-FS nanocomposite are thermally more stable in contrast to the pristine PC due to the interplay between PC chain and the silanol group of fumed silica when melt blended at 280 °C^28, 29^. In contrast to the thermal stability and high elastic modulus, the developed composites have manifested lower storage modulus and glass transition temperature by the virtue of the plasticizing effect of fumed silica which augmented segmental moment of PC chain at lower temperature. We envisioned that though, the interfacial zone between inorganic and organic groups possess physical interplay and well-dispersed system, the bulk properties of polymer nanocomposites also depend on the physical condition of nanofiller (amount of moisture at silica surface, physical dimension and the attribute of filler at the glass transition temperature of the polymer matrix).

## Materials and Experimental Details

**Figure 1:**
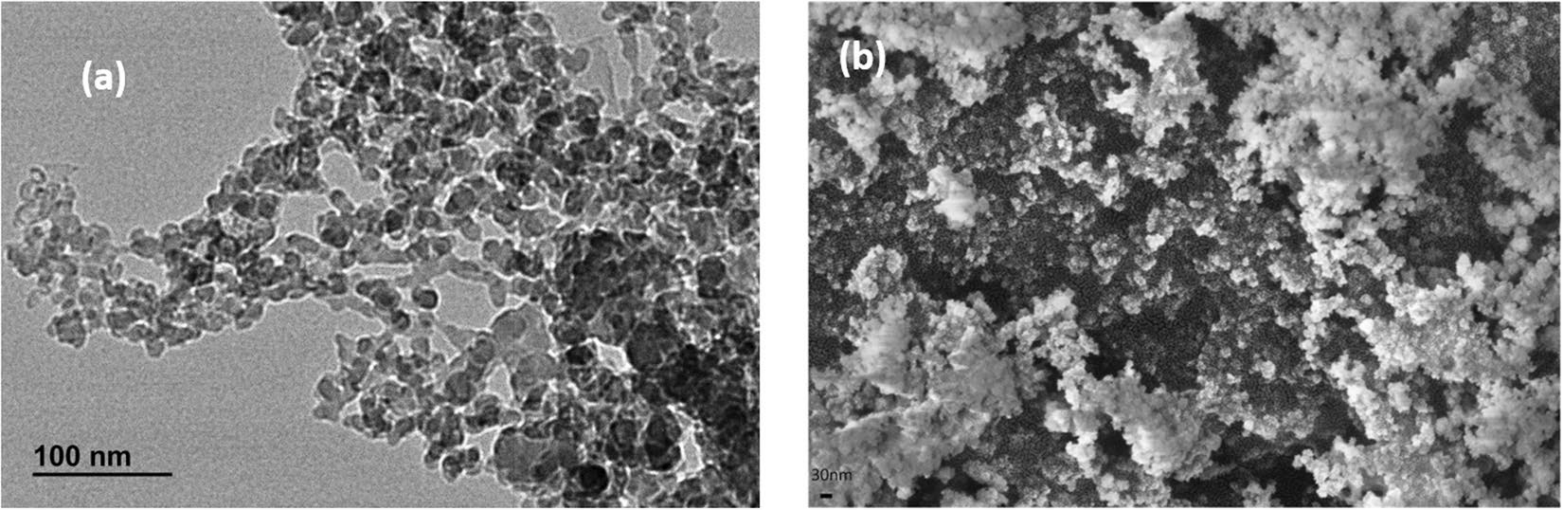
(**a**) TEM image of fumed silica Nanoparticles and (**b**) FESEM image of fumed silica.

**Figure 2:**
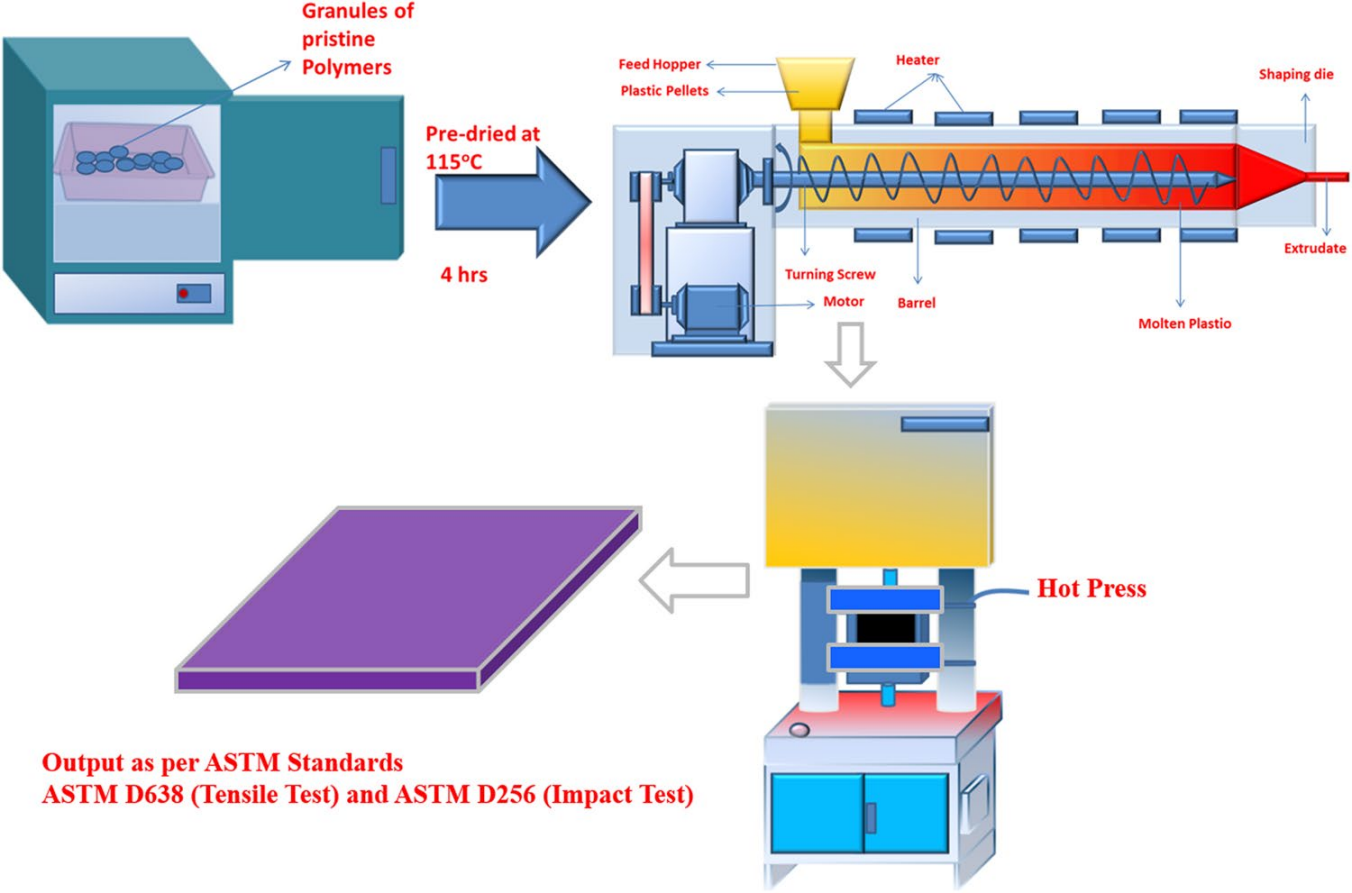
Fabrications of PC-FS Nanocomposite via melt Blending and Hot Press Technique.

Polycarbonate (PC) (MFI = 10.5 g/10 min, viscosity 22 cp, LEXAN grade 143 R, SABIC Innovative Plastics India Pvt Ltd, India) and fumed silica (FS) with the size of 7-14 nm (Sigma-Aldrich Corp. USA) were used for the preparation of the samples. The size distribution of fumed silica has been illustrated in Fig. 1(a) and (b) ^22^. All reagents involved were of analytical grade and were used without any further purification. Polycarbonate pellets were dried in a vacuum oven for 4 h at 115 °C for removing moisture and other volatiles, while fumed silica was dried at 120 °C for 8 hours to dehydrate its surface. PC-FS nanocomposite were fabricated by melt blending of 5 weight % of fumed silica in polycarbonate matrix via LabTech twin screw extruder (Thailand) with the melt temperature of 280 °C at screw speed of 30 rpm. The extruded fibers were further pelletized via LabTech pelletizer. Dynamic Mechanical analysis, Tensile Test and Impact Test samples were prepared in accordance with ASTM standard (ASTM-D4065, ASTM-D638 and ASTM-D256 respectively) by employing a hot press technique followed by cooling at ambient temperature under the hydraulic press. The entire process of sample preparation has been illustrated in Fig. 2.

## Results and Discussions

**Figure 3:**
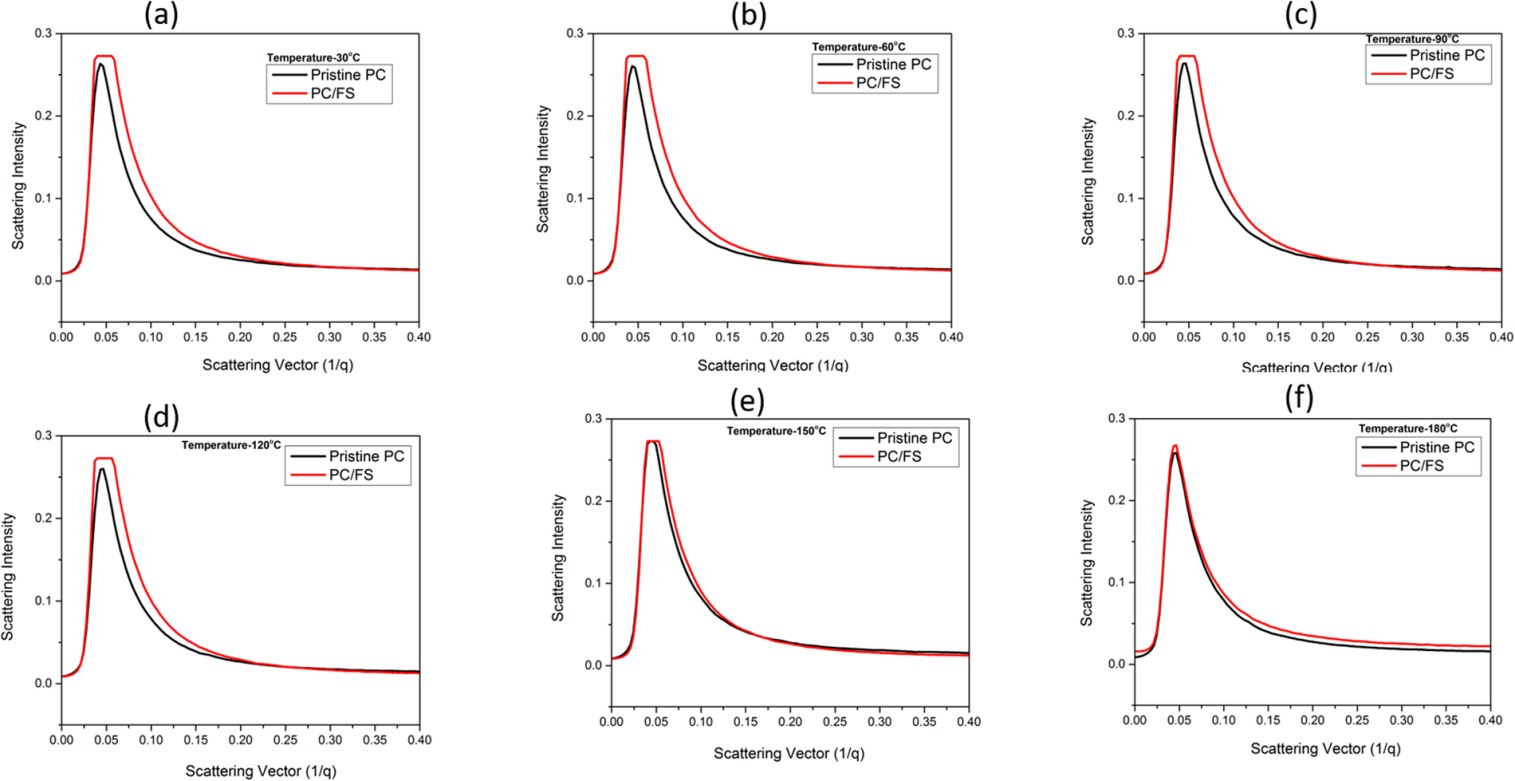
(**a**) Temperature assisted SAXS spectra of pristine polycarbonate PC-FS Nanocomposite at various temperatures (**a**) at 30 °C (**b**) at 60 °C (**c**) at 90 °C (**d**) at 120 °C (**e**) at 150 °C (**f**) at 180 °C.

As stipulated earlier, polycarbonate endures limited thermal degradation reaction below 250 °C in evacuated system that lead to the random chain scission of the polymer^13, 29, 30^. As expected, we have gleaned analogous result in SAXS spectra, as depicted in Fig. 3. For all the temperature, the first peak at lower q region can be associated to the lamellar periodicity produced by polycarbonate^31^. The intensified diffraction peaks in the case of polycarbonate can arise either due to the intramolecular atomic distances, intermolecular atomic distances or local ordered structure. It has been widely exploited that crystallization of polycarbonate is a slow process over a restricted temperature range^32–34^. The Bragg diffraction peak for PC-FS nanocomposite demonstrated the consistent diffraction peaks for all the temperature, which can be associated with the augmented density packing of polycarbonate when reinforced with high concentration of FS. In contrast to the Bragg diffraction peak of PC, SAXS spectra of composite rendered widened diffraction peak, which is attributed to the disordered structural orientation of fumed silica domain in polymer matrix due to the augmented density packing. It has been observed that the incorporation of FS in PC matrix advances the segmental movement at 150 °C as evident in Fig. 3(e). It is acclaimed that preheating of fumed silica is a preeminent process parameter due its tendency to absorb water from the air moisture via hydrogen bonded water or physical absorption^35^. G. J. Young demonstrated that physical adsorption of water vapour on silica surface is restricted to the vicinity of silanol site and complete surface dehydration is not possible even heating silica at 180 °C^36^. He has postulated that considerable surface strain is generated at the silica surface when heated at lower temperatures due to the condensation reaction which forms silica-oxygen bond and water. Therefore, strained surface possesses a high tendency of water absorption when exposed in ambient environment during the processing condition. Though, chemisorption behaviour of water vapour can be curtailed at 400–500 °C to obtain the complete dehydrated silica surface but higher temperature heating forced the silanol group to relinquish the silica surface. In this context, we have anticipated that fumed silica in PC matrix contains water molecules due to the chemisorption attribute of water vapour even silica was preheated at 180 °C. When nanocomposite was exposed to the temperature beyond 150 °C (Fig. 3e), the absorbed moisture of fumed silica rendered plasticizing effect of the PC matrix which advances the segmental movement of PC chain. The transition of stable disordered PC orientation to molecular dynamism resulted in analogous PC diffraction peak in developed composite as delineated in Fig. 3(f). The change in the scattering vector region at 150 °C and 180 °C can be corroborated to the glassy dynamics of interface layer which further exhibited reduced glass transition temperature (Table 1).

Polycarbonate is considered as crystallizable polymer but in contrast to other crystallizable polymer, the process of crystallinity in polycarbonate is very slow. Therefore, in our study we do not speculate any changes in the amorphous characteristics of PC and its developed composites. The electron density profile has been regarded as prominent tool to enumerate the temperature dependence amorphous/crystal ratio in SAXS spectra. This hypothesis was utilized by Men *et al*. to evaluate the linear crystallinity of cold drawn polyethylene^37^. They have cautiously mentioned that amorphous or crystalline thickness from correlation function can be obtained only when the prior knowledge of the polymer crystallinity is manifested. In order to intuitively obtain, the long-range period related to the position of scattering peak, Bragg’s law has been employed^38, 39^.

$${d}_{ac}=\frac{2\pi }{{q}_{\max }}$$

d_ac_ is long spacing obtained from Bragg’s law (d_ac_ = d_a_ + d_c_, where da and dc are the average thickness of amorphous and crystalline region as illustrated in electron density profile of polycarbonate and PC/FS composite).

**Figure 4:**
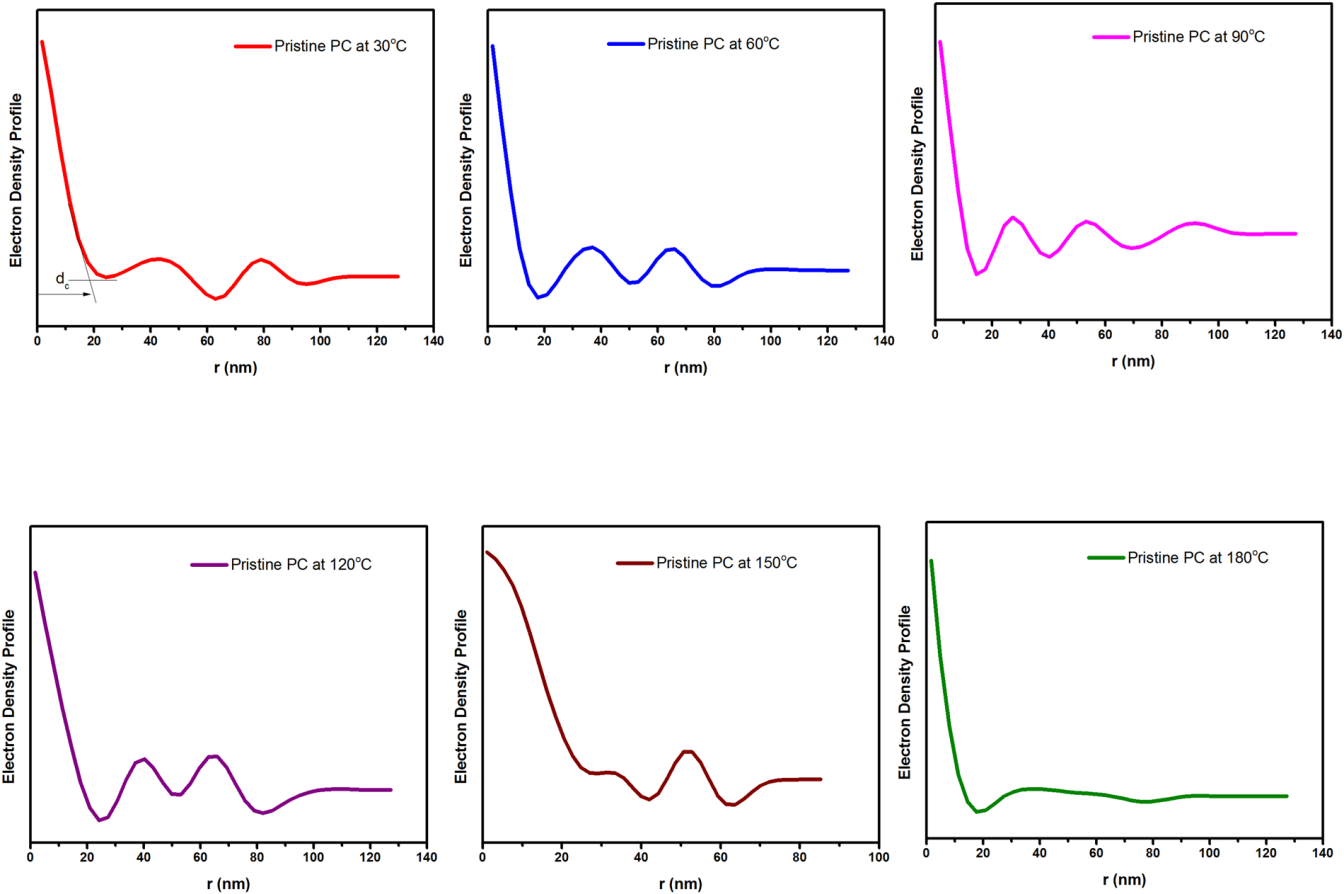
Electron density profile of pristine polycarbonate at various temperatures.

**Figure 5:**
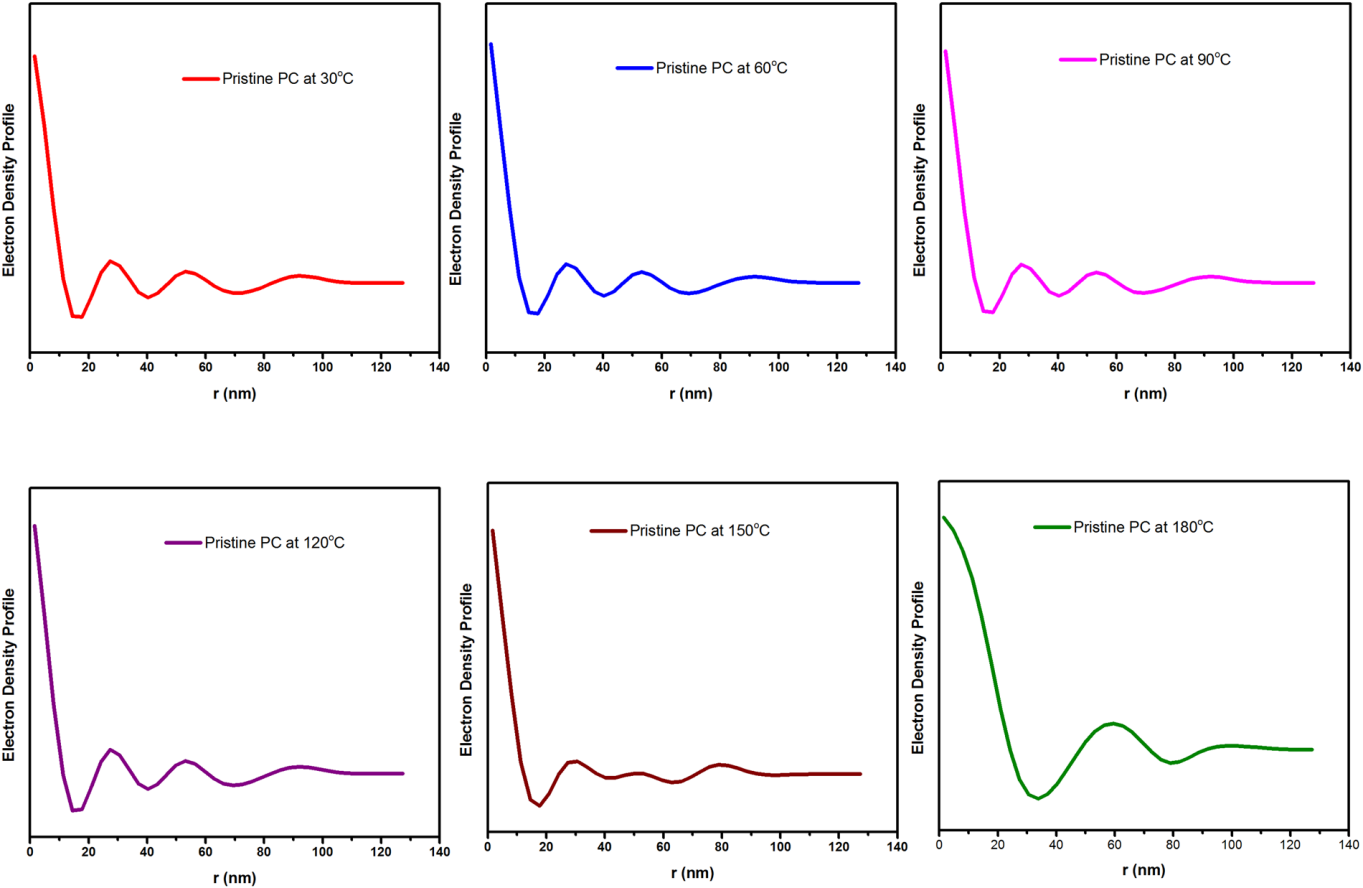
Electron density profile of pristine polycarbonate/Fumed silica composite at various temperatures.

As evident from Table 1 and Figs 4 and 5, the amorphous region in PC and developed composite has not been significantly altered during the exposure of *in-situ* SAXS spectra. The crystalline region gleaned in the electron density profile is very small to render any change in the amorphous region of polycarbonate.

**Table 1:** Structural evaluation of pristine polymer and composite obtained from electron density profile.

Temperature (°C)	Long Range spacing for PC (d_ac_pc) (nm)	**A**morphous region **d**_**a**_(pc)(nm)	**%** amorphous region	**L**ong Range spacing for Composite (dcom)	**A**morphous region **d**_**a**c_(com)	**%** amorphous region
30	146.12	128.12	87.59	136.59	119.59	87.55
60	146.12	128.12	87.59	136.59	119.59	87.55
90	146.12	128.12	87.59	136.59	119.59	87.55
120	146.12	125.12	85.62	136.59	119.59	87.55
150	149.59	127.59	85.29	136.59	119.59	87.55
180	149.59	131.59	87.96	136.59	113.59	83.16

**Figure 6:**
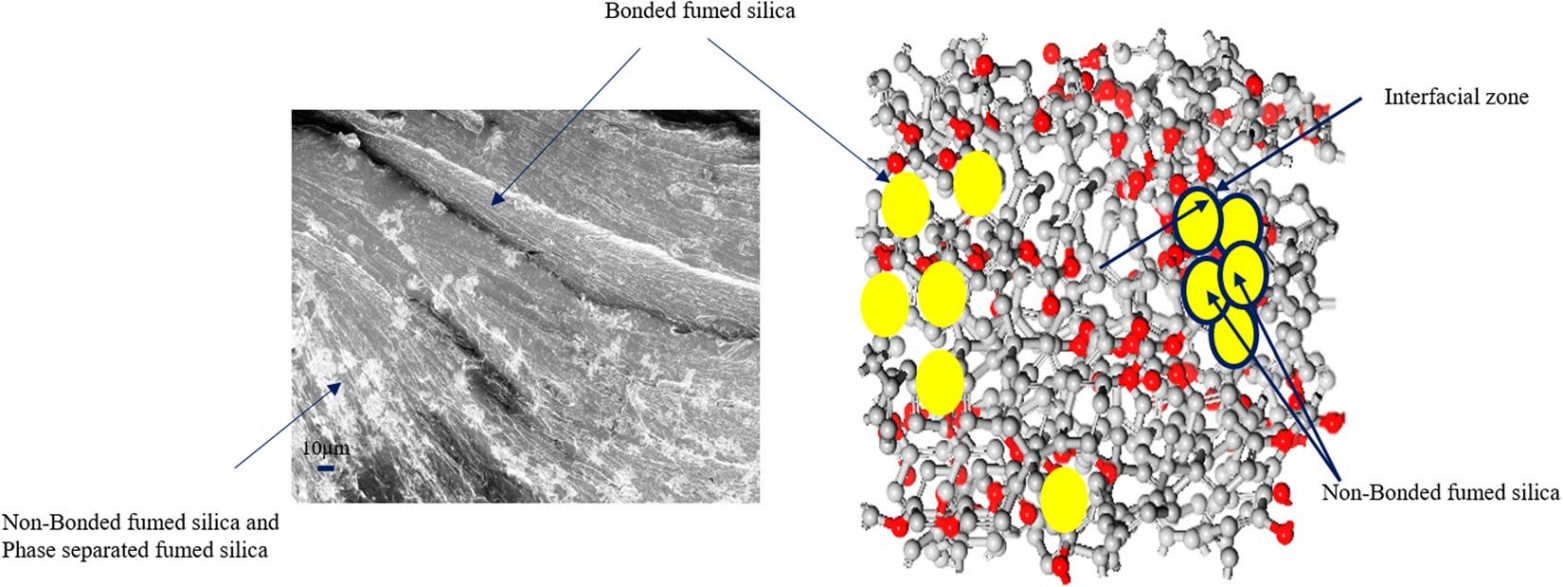
(**a**) FESEM image of fractured surface of polycarbonate and (**b**) Elucidation of phase separation and agglomeration of fumed silica in PC matrix.

Though, polycarbonate evidenced hydrogen bonding interaction with silica surface containing hydroxyl group during melt blending (confirmed by FT-IR spectra Fig. 3), phase separation and agglomeration is also a critical feature of blending at high concentration of nano-reinforcement^28, 29^. Sanchez *et al*. delineated that inorganic and organic nanocomposite can be classified in two categories where in class I, organic and inorganic components are embedded and cohesion in whole structure is obtained via weak forces (Hydrogen bonding, van der Wals forces) while class II consists of two phases linked together through strong chemical bond (covalent bond. Iono-covalent bond)^40^. In addition, X. Hao *et al*. illustrated that incorporation of silica nanoparticles in PLA exhibits two type of interaction: particle-polymer interaction and particle-particle interaction and further particle and polymer interaction evolve through the physical adhesion between polymer and particle in addition to the hydrogen bonding^41^. In this context, we speculated class I interaction between polycarbonate and fumed silica nanofiller that consists of two type of interaction where one could be associated to those silica moieties that have been physically interacted with the PC chain during melt blending at 280 °C. While other interaction could be allied to the non-contact bonded silica particles which emanated from phase separation and agglomeration containing FS silica-rich phase and PC rich phase separated by interfacial region^27, 42^. In this abstraction phase separation can be defined as the combination of continuous polymer matrix and inhomogeneous distributed fumed silica phase while agglomeration evolved due to the assembly of aggregates of primary fumed silica particles^43^. The aggregates of the primary fumed silica is evolved through the fusing of 10 to 30 spheres during the burning of silicon tetrachloride which further creates reversible mechanical entanglements which is called as agglomerates of fumed silica^44^. When those entangled fumed silica particles are utilized in melt blending process, they further develop agglomeration and creates a separate phase in composite as elucidated in Fig. 6(a) and (b). It is clearly evident from the fractured surface of composite that some fumed silica moieties are loosely bonded with PC matrix which resulted in two phase system while some moieties are in the continuous phase with polycarbonate matrix.

**Figure 7:**
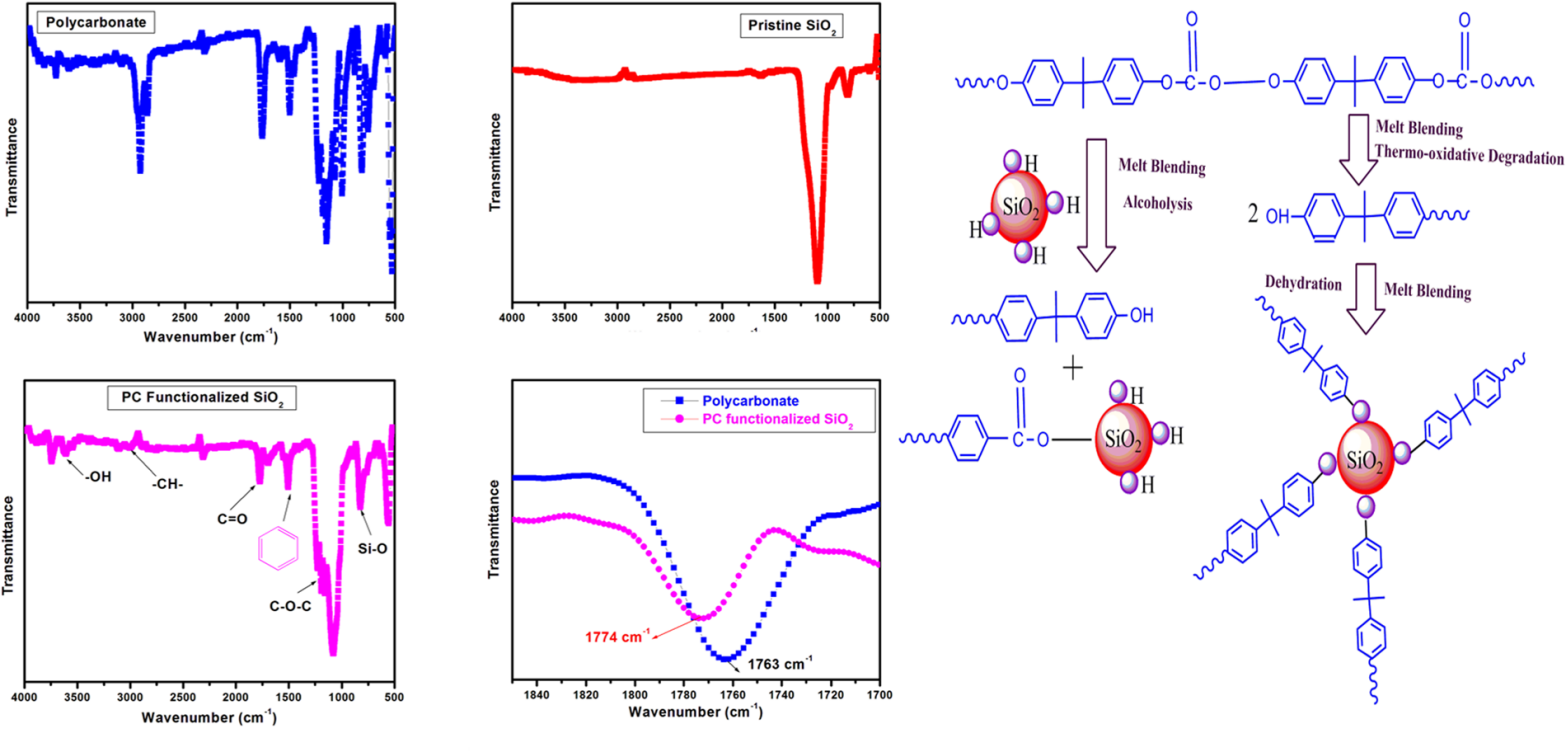
FT-IR analysis of (**a**) Pristine Polycarbonate (**b**) Pristine SiO_2_ (**c**) PC functionalized Fumed Silica (**d**) Elucidation of blue shift of C=O peak. (**e**) Schematic Representation of Functionalizing fumed silica by Degraded PC chain.

The former interfacial interaction between polycarbonate and fumed silica during melt blending has been confirmed via FTIR spectroscopy technique as elucidated in Fig. 7 and Table 2. The stretching vibration gleaned at 1774 cm^−1^ elucidated blue shift of C=O peak in functionalized SiO_2_ in contrast to the pristine PC that substantiate the alcoholysis reaction between silanol group of fumed silica and carbonyl group of PC^28^. These abstractions apparently render that the main active group to form thermally stable interfacial zone, is the formation of Si-O-C bond due to the interaction between the nano Fs and carbonate group in PC chain as exemplified in Fig. 7(e). Therefore, we envisioned that molecular dynamism of the PC/FS composite emerges from the combined effect from the segmental mobility and plasticizing effect of FS, attributed to the physically grafted FS and formed interfacial layer of phase separated region in PC matrix^27^.

**Table 2:** Bond analysis of various stretching vibrations obtained in FT-IR spectra.

Wave Number (cm^−1^)	Stretching Vibration
2972, 2876	C-H
1779	C=O
1510	Phenyl
1226, 1196, 1164	C-O-C

**Figure 8:**
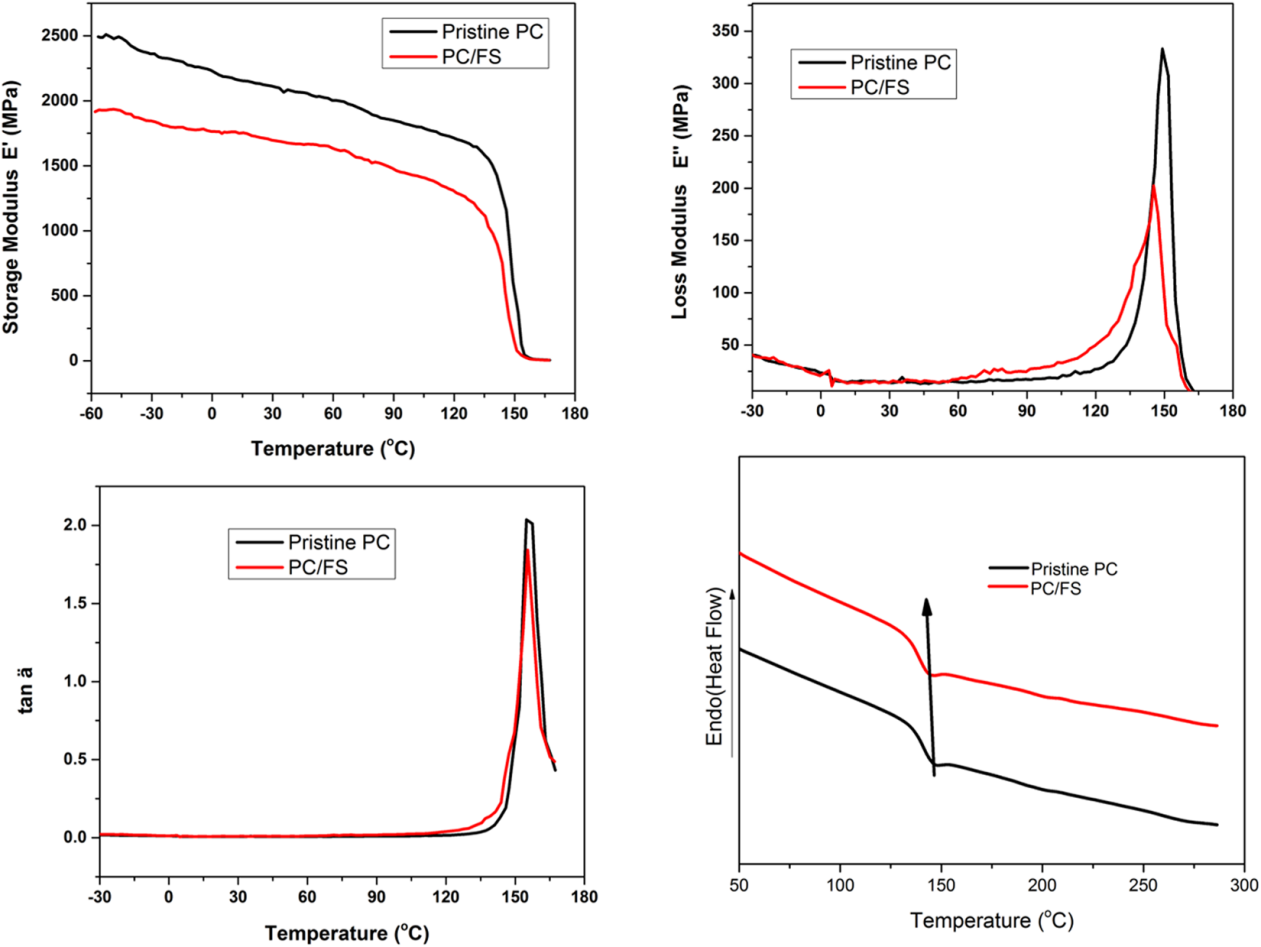
(**a**) Storage Modulus of Pristine PC and PC-FS composite (**b**) Loss Modulus of Pristine PC and PC-FS composite (**c**) Damping behaviour of Pristine PC and PC-FS composite (**e**) DSC curve of Pristine PC and PC-FS composite.

The confirmation of molecular dynamism of PC matrix in the presence of fumed silica under the cyclic deformation and temperature condition has been further substantiated by dynamic mechanical analysis (DMA). The storage modulus characteristics obtained in DMA exemplify the response of any materials against the applied cyclic load i.e. load required to introduce deformation in the system^45^. The value of storage modulus is corroborated to the stiffness or elastic nature of the polymer^46^. As evident in Fig. 8(a), the storage modulus of PC-FS composite is dramatically reduced compared to the pristine PC which is attributed to the enhanced stiffness and reduced molecular mobility of the composite due to the disrupted stoichiometry ratio in the presence of FS (High packing density). The obtained result in DMA illustrated analogous result acquired in SAXS. The stiff behaviour of polymer nanocomposite (impact = 337.20 ± 10 j/m) demonstrated 40% reduction in its impact property at ambient temperature in contrast to the pristine polymer (impact = 843 ± 10 j/m).

Figure 8(b) delineate the heat loss modulus parameter of DMA which illustrates the ability of a material to dissipate energy under the extreme loading conditions. It has been noted that the loss modulus of PC-FS composite exhibited gradual increase till the glass transition temperature of the composite. The increase in the loss modulus is pertinent to the increased molecular friction in the presence of fumed silica but beyond the glass transition temperature the exponential reduction in the response of composite can be associated to the plasticizing effect of moisture available at the surface of fumed silica. Since, the availability of water molecules on the surface of fumed silica results in plasticizing effect, the α transition in PC composite was observed at lower temperature (145 °C) which impart enhanced segmental movement and reduced glass transition temperature^46–48^. The change in the glass transition temperature has been also observed in Tan Ϩ and DSC as demonstrated in Fig. 8(c and d). Masenelli-Varlot *et al*. have assigned the diminishing peak of Tan Ϩ to the plasticizing effect of nanofiller with the increasing brittle nature of the composite^49^. They have concluded that loss factor is associated with the response of system components against the deformation energy applied to the complete composite systems. Therefore, the filler geometry, their orientation in polymer matrix and their crystalline structure presumably affects the dynamic response of polymer nanocomposite.

**Figure 9:**
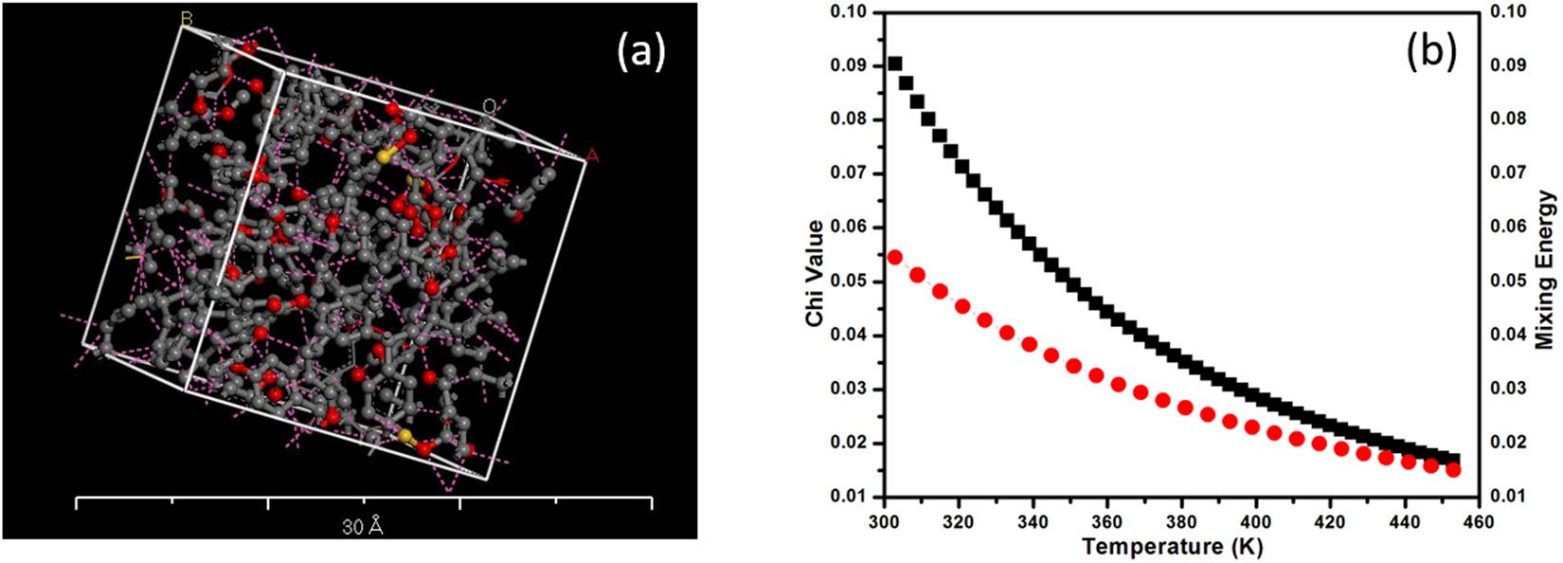
(**a**) Amorphous unit cell of fumed silica/PC nanocomposite (**b**) Interaction Parameter and free energy of mixing for fumed silica/PC nanocomposite obtained from DPD analysis.

As stated earlier, the performance of a composite system is critically controlled by the interfacial characteristics between polymer matrix and reinforced nanofillers. In this context Flory-Huggins interaction parameter (χ) and free energy of mixing are influential parameters to examine the interfacial interaction between polymer matrix and nanoparticles. Several methods have been employed for the estimation of Flory-Huggins interaction parameter (χ) between polymer-polymer, polymer-solvent and solvent-solvent systems^45, 50^. Here, we have tried to outline the window of interaction parameter and free energy of mixing via Dissipative Particle Dynamics (DPD) methodology of Accelrys Material studio. We have first delineated the optimized amorphous unit cell of PC/FS nanocomposite with the dimension of 20 × 20 × 20 by utilizing forcefield as elucidated in Fig. 9(a). The PC/FS melt blended polymer nanocomposite evidenced lower interaction parameter and mixing energy as demonstrated in Fig. 9(b) presumably attributed to the enhanced cohesive energy due to the identical functional units of polymer chain and modified nano silica surface, which has been endowed to be further improved with the augmentation of temperature^51, 52^. The functionalized moieties of SiO_2_ act as a sticker that bind PC backbone and thus constitutes long chain structure which further promotes the strong interfacial interaction between both the systems. Consequently, the improved interfacial adhesion has been broadly reported to impart the thermal stability to the polymer matrix compared to the conventional polymer nanocomposite due to the reduced molecular mobility^53, 54^. This abstraction apparently support the hypothesis developed in FTIR analysis which explains the interaction between silanol group of fumed silica and polycarbonate chain. We have speculated that the positive value of the interaction parameter and free energy of mixing can be associated to the non-bond interaction between fillers and polymer matrix that lead to the evolution of electrostatic and Van Der Wall interaction^55^.

**Figure 10:**
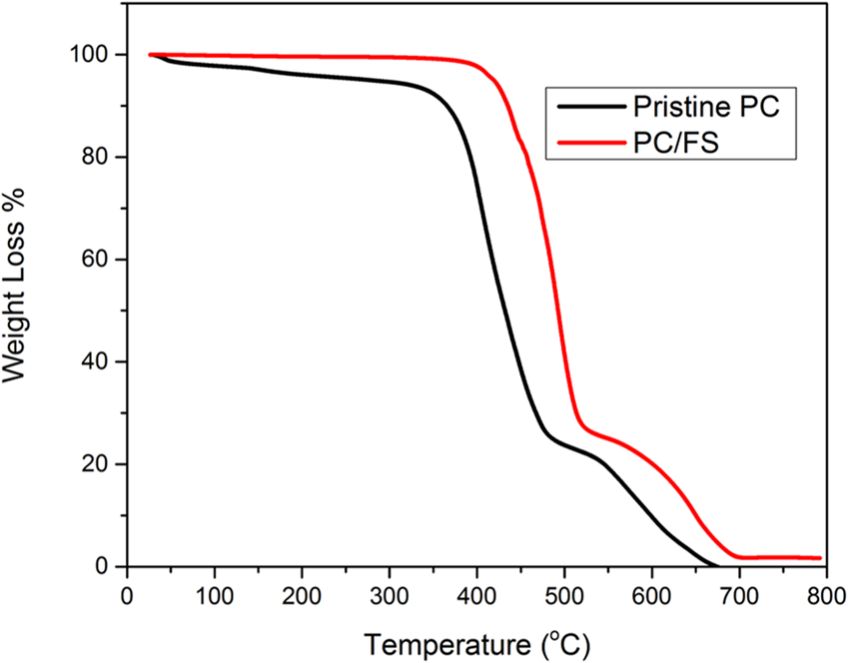
Thermal degradation behaviour of PC and PC-FS.

Though, the molecular orientation and absorbed moisture on fumed silica surface largely exploit the dynamic characteristics of composite but the incorporation of fumed silica yield improved bulk thermal stability in terms of degradation characteristics and elastic modulus as exemplified in Fig. 10. It is apparent from Table 3 that the onset of degradation temperature (T_onset_), degradation temperature at 50 wt% weight loss (T_0.5_) and the degradation temperature at maximum weight loss are significantly increased. The augmented thermal stability by incorporating inorganic fillers is commonly ascribed to the barrier network formed by the nanoparticles and the hindrance offered by char in transferring the volatile products and heat during degradation process. Jang and Wilkie have reported that inorganic additives are also involved in the degradation process in addition to the barrier network hypothesis. They have reported that silica nanoparticles can capture the radicals produced by the scission of isopropylidene and limit their mobility at higher temperature which leads to the improvement in T_onset_^56, 57^.

**Table 3:** Thermal stability parameters of pristine PC and PC-FS composite.

Sample	T_onset_(°C)	T_0.5_(°C)	T_max_(°C)
Pristine PC	336	431	485
PC-FS Composite	413	493	526

## Conclusions

In summary, we have delineated the feasibility of utilizing temperature assisted *in-situ* SAXS and FT-IR analysis for characterizing the thermal and structural stability of polycarbonate and polycarbonate decorated fumed silica nanocomposite. It has been demonstrated that when the fumed silica was melt blended with polycarbonate, resulted in the interaction between silanol group of silica surface and polycarbonate chain which renders enhanced thermal stability ascertained by *in-situ* SAXS. We have speculated that the formation of interfacial zone between nanofiller and polymer matrix imparts improved thermal stability, lower interaction parameter and reduced free energy of mixing. The positive value of both the interfacial parameters was corroborated to the evolution of electrostatic and Van Der Wall interaction between fumed silica and polymer matrix. We envisioned that this abstraction can be effectively utilized to contemplate the structural and thermal stability of polymer nanocomposite which are extensively exploited in numerous application like electronic appliances, automobile, architecture and aerospace.
